# Low plasma cortisol and fecal cortisol metabolite measures as indicators of compromised welfare in domestic horses (*Equus caballus*)

**DOI:** 10.1371/journal.pone.0182257

**Published:** 2017-09-08

**Authors:** Jodi Pawluski, Patrick Jego, Séverine Henry, Anaelle Bruchet, Rupert Palme, Caroline Coste, Martine Hausberger

**Affiliations:** 1 Institut de recherche en santé, environnement et travail (Irset), U1085 INSERM, Université de Rennes 1, Rennes, France; 2 Université de Rennes 1, UMR 6552 CNRS Ethologie Animale et Humaine, Station Biologique de Paimpont, France; 3 University of Veterinary Medicine, Department of Biomedical Sciences, Vienna, Austria; 4 CNRS, UMR 6552 Ethologie animale et humaine, Université de Rennes 1, Rennes, France; Radboud Universiteit, NETHERLANDS

## Abstract

The hypothalamic-pituitary-adrenal (HPA) axis response to chronic stress is far from straight forward, particularly with regards to animal welfare. There are reports of no effect as well as both decreases and increases in cortisol after chronic stressors. Therefore, the first aim of the present study was to determine how measures of compromised welfare, such as chronic pain and haematological anomalies, related to cortisol levels in domestic horses (*Equus caballus*). Domestic horses are an informative model to investigate the impact of chronic stress (due to environment, pain, work, housing conditions…) on the HPA axis. The second aim was to determine whether levels of fecal cortisol metabolites (FCM) may be used as an indicator of welfare measures. The present study used fifty-nine horses (44 geldings and 15 mares), from three riding centres in Brittany, France. The primary findings show that horses whose welfare was clearly compromised (as indicated by an unusual ears backward position, presence of vertebral problems or haematological anomalies, *e*.*g*. anaemia) also had lower levels of both FCM and plasma cortisol. This work extends our previous findings showing that withdrawn postures, indicators of depressive-like behavior in horses, are associated with lower plasma cortisol levels. We also found that evening plasma cortisol levels positively correlated with FCM levels in horses. Future research aims to determine the extent to which factors of influence on welfare, such as living conditions (e.g. single stalls versus group housing in pasture or paddocks), early life factors, and human interaction, act as mediators of cortisol levels in horses.

## Introduction

Animals confronted with a physical or emotional stressor rely on an intricate interplay between many biological systems (behavioral, autonomic, neuroendocrine and/or immune) to elicit responses to cope with that stressful situation [1–3]. Among stress reactions, the activation of hypothalamic—pituitary—adrenocortical (HPA) axis has been extensively studied [4]. However, our understanding of how cortisol, the main hormone of the HPA system, is related to chronic environmental conditions related to housing, work versus rest, and social interactions, remains to be determined [4–8].

Although HPA axis activation is well described in response to acute stress, its interpretation in cases of chronic stress is far from straight forward [4–8]. Indeed, poor environmental conditions (*e*.*g*. social deprivation, social conflicts, confinement) appear to have different effects on the HPA axis. Thus, baseline cortisol levels and/or HPA axis reactivity of animals subjected to chronically stressful environmental conditions have been reported **(i)** to remain similar (*e*.*g*. socially isolated cattle and indoor versus outdoor housing of sheep [9–11]), **(ii)** to increase (*e*.*g*. pigs experiencing noise exposure [12, 13]; reared under poor environmental conditions [14]; or confined [15–17]), or **(iii)** to be depressed (socially isolated cattle [18]; bulls subjected to individual confinement after previous group housing: [19] and confined calves [20]; female European starlings submitted to repeated exposure to predators, loud sounds or novel objects [21]). Reports linking chronic stress and HPA function in humans are also contradictory, with some evidencing increased cortisol levels, and others the opposite [7, 8]. These differences may be due to the type of the stressor(s) as well as the duration of the stressor(s).

Moreover, the effects of chronic stress on physiological data and other responses (such as behavior) can also be contradictory. For example, the plasma cortisol levels of pigs submitted to unpredictable and inescapable electric shocks for a month were similar to those of control animals that had received no shocks [22]. However, their behavior (agitation followed by inhibition) suggested that they were still stressed by the situation [22]. Houpt *et al*., (2001) found no differences between the cortisol levels (baseline plasma levels and HPA sensitivity to ACTH) of pregnant mares confined for six months and given only limited exercise (one 30 minute period in a paddock every 14 days) and those of mares living under the same conditions, but exercised (for 30 minutes) daily [23]. However, confined mares increased their locomotion when released from confinement compared to the daily-exercised mares, indicating a behavioral response to exercise deprivation. Comparing the impact of one month of two types of housing at weaning, it was found that individually housed horses (with a high level of confinement and social isolation) showed more vigilant behavior in their box than did horses housed in pairs [24]. However, no significant differences in basal plasma cortisol levels could be attributed to housing condition, and the response in the corticotrophin-releasing factor (CRF) challenge test revealed no clear treatment differences [24]. We have also recently shown that there is no relationship between levels of plasma cortisol or fecal cortisol metabolites (FCM) and stereotypic behaviors, *i*.*e*. repetitive behaviors induced by frustration, repeated attempts to cope and/or brain dysfunction, in riding school horses [25] but ‘withdrawn’ horses have *lower* plasma cortisol levels [26]. Consequently, there appears to be disconnect between long-term exposure to poor environmental conditions on cortisol levels and behavioral outcomes related to overall welfare.

In this study on horses, we wanted to 1) explore the relationship between cortisol levels and additional measures of welfare that may be affected by chronic stressors and 2) determine if fecal cortisol metabolite levels could be used as an appropriate indicator of plasma cortisol and be related to welfare measures. Different definitions have been given for animal welfare, such as a balanced physiological state [27], the satisfaction of the behavior (“psychological “needs, [28]) or the absence of negative emotions and ideally the presence of positive emotions [29]. Many authors emphasize the need for an animal-centered approach, i.e. based on physiological, sanitary and/or behavioral measurements [30]. Domestic horses (*Equus caballus*) are an informative model to investigate the impact of chronic stress (due to environment, pain, work…) on the HPA axis, for a number of reasons: First, horses commonly experience environmental conditions challenging their welfare, and they are highly sensitive to these environmental factors (*e*.*g*. by developing stereotypies, reviewed in [31]). Second, domestic horses are extremely interesting models as they exhibit visible signs of impaired welfare when living in inappropriate conditions. These include abnormal repetitive behaviours (including stereotypies [31–35], posture alterations [36–38], behavioural problems (*e*.*g*. aggressiveness; [26, 39] and apathy [26, 40, 41]). Third, these visible (behavioural and/or postural) signs add to commonly reported vertebral [37–39, 42–45]) and health disorders [34, 46, 47]. The factors impairing welfare are related to social, spatial and feeding restrictions [35, 47–49]; inappropriate diet (*i*.*e*. high energy diet; [50]) and working conditions [32, 37–39, 51–53]. Work has also been shown to influence the chronic emotional state of show horses [54]. Thus horses are a valuable model to understand how multiple extrinsic and intrinsic factors may act to affect long term function of the HPA system.

We used a multidimensional approach to investigate the relationship between baseline cortisol levels on the one hand, and selected indicators of chronic compromised welfare, including health-related (vertebral problems, haematological disorders), and postural (ears-backward position) indicators. As mentioned above, a previous study has already shown that apathetic horses have lower cortisol levels while no correlation was found with stereotypic behaviours [25, 26]. Horses came from riding schools, meaning that tests of HPA axis reactivity (involving exogenous administration of substances) were not possible. Consequently, we used cortisol baseline levels measured by both classical (*i*.*e*. blood sampling) and non-invasive methods (feces sampling) validated for horses measuring fecal cortisol metabolites (FCM) [55, 56]. Such non-invasive methods offer advantages over traditional invasive methods, as samples can be collected easily and can provide a better assessment of long-term glucocorticoid levels than plasma samples [2, 57].

## Materials and methods

### Subjects

All our experiments complied with current French laws related to animal experimentation and were in accordance with the European directive 86/609/CEE. The project was approved by the local Ethics Committee in Animal Experiments at the University of Rennes 1. Fifty-nine horses (44 geldings and 15 mares), from three riding centres in Brittany, France, were tested. Sixty-eight percent of the horses were French Saddlebreds, equally distributed among the centres. Other horses belonged to a variety of breeds or were unregistered animals. Their ages ranged from 5 to 20 years old ($\bar{X}\;=\;11.9\pm 3.5$). Activities and housing conditions in these centres were similar. In all cases, horses were kept singly in 3 m x 3 m individual straw-bedded boxes. Each box was cleaned once a day (in the morning). Animals were fed industrial pellets three times a day and hay was provided *ad libitum* once a day. Each box was equipped with an automatic waterer. Horses worked in riding lessons for 4–12 hours a week, with at least 1 free day each week (closing day). Riding lessons involved children and teenagers and were mainly related to indoor (instruction) and outdoor activities, including a few competition activities. Riding techniques corresponded to the usual English style and could differ slightly between centres [38, 58]. Over the course of the study, the observed horses were not submitted to any particular veterinary treatment apart from routine procedures such as deworming or vaccines. Order of sampling was chiropractic analysis, fecal sample collection and serum collection. These measures were separated by 3 to 7 days. Additional work on a subset of these animals is published [25, 26, 39].

### Plasma cortisol

For plasma samples, blood was collected from the neck vein of each horse while in its box as previously described [26]. All blood samples were obtained in presence of a veterinarian. To do this each horse was lightly restrained by one unknown (to the horse) experimenter, gently petted and systematically given a food reward (one sugar lump) at the end of the blood sampling. The total duration of this procedure, from opening the box door to leaving the box did not exceed one minute. Seven millilitres of blood were collected in heparinised polypropylene tubes (BD Vacutainer^®^). Samples were kept in crushed ice until centrifugation (with a maximal delay between sampling and centrifugation of 15 minutes) and then aliquots of plasma were immediately placed on dry ice and stored at -20°C for further processing. Blood samples were collected three times from each subject: once between 18:00 and 19:00 after a day’s work, once between 18:00 and 19:00 after a day’s rest and once between 08:00 and 09:00 (around one hour after their first meal of industrial pellets). We chose these schedules so as to take into account both circadian variations [59–64] and a potential impact of working activities (physical exercise, and also agitation and noise in the stable during daytime) on cortisol levels. Samples were taken in spring and in early summer 2007.

Plasma cortisol levels were measured using Immunotech kits for cortisol determination (Beckmann and Coulter). The kit was modified for equine plasma as follows: 1) the quantity of plasma per dose was 25 μL instead of 50 μL; 2) a two-hour preliminary incubation at 20°C between plasma and antibodies was added; 3) we used two standard curves: the first with increasing cortisol contents in buffer (as indicated by the manufacturer) and the second with increasing cortisol contents in equine plasma (from pooled data for controls totaling less than 2 ng/mL of cortisol). When data did not fit these two standards, they were discarded. These modifications produced linear curves (log B/Bo) between 2 ng/mL and 300 ng/mL. A good linearity was observed for dilution or overload experiments. The statistical reproducibility variation coefficient was 1.37% and the sensitivity was 2 ng/mL. Plasma concentrations of cortisol measures were previously reported in relationship to withdrawn postures in Fureix *et al*. (2012).

### Fecal cortisol metabolites (FCM)

To determine the relationship between FCM and plasma cortisol levels, and FCM and welfare measures, fresh fecal samples were collected within 1 minute of defecation, directly from the bedding by an experimenter using sterile gloves. Samples were collected between 12:00 and 13:00, three times for each horse: twice 24h after a day’s work and once after a day’s rest (again taking into account the potential impact of working), based on previous work in ponies [65]. Samples were frozen at −20°C until further analysis. Feces were extracted [56] and then analysed by an 11-oxoetiocholanolone enzyme immunoassay (EIA) as described by Palme and Möstl (1997). Increases in plasma cortisol are evident in the feces with a delay time of about 24 hours and depending upon frequency of defecation represents pooled HPA activity over a window of ~2 hours [55] where the method was validated for horses. 11,17-dioxoandrostanes was the metabolite measured as per [25, 55].

The two fecal measures after a day’s work were averaged to give one measure and indicate HPA activity during work. Outliers more than 3SD above or below the mean were removed. This occurred once due to technical error with FCM after a day’s rest.

### Welfare state indicators

To determine general welfare state and how welfare may be related to cortisol levels, some indicators such as ear positions (while feeding, [36, 39, 42, 66], vertebral disorders [37, 39], and haematological data were assessed.

#### Ear position

In horses, backward ear position is commonly associated with negative emotional states, such as pain or a negative cognitive bias [34, 67, 68]; or during agonistic interactions [69]. Ear position for each horse was noted at 15 minute intervals and on two different rest days until 10 ear positions were obtained per horse according to the method used by Lesimple et al. [42, 43]. Three positions were defined in accordance with previous studies on sheep and horses [67, 70]: axial (perpendicular to the head—rump axis), forward (tip of the ear towards the front at an angle of more than 30° from the perpendicular) or backward (tip of the ear towards the back at more than 30° from the perpendicular). In order to have homogeneous conditions, the observer recorded ear positions only if the horse did not show any reaction (*i*.*e*. no change in behaviour) when being observed, and in a single context: the horse had to be foraging on hay or straw or grass, head down as it has been shown to be the most reliable context [39]. For the riding school horses, observations were made in the stables in calm conditions (outside feeding times, day off, 2 to 5 pm, no wind). The experimenter walked slowly and regularly (1 step/sec) along the boxes and noted for each horse the instantaneous ear position. The observer approached slowly each box so as to be able to see ear positions through the feeding opening or the box window while remaining at a distance. This quiet approach did not elicit any of the strong reactions observed after appearing suddenly at the box door [71].

The observer then resumed walking along the midline to the next box. The percentage of scans in each ear position was calculated for each horse. Further analyses categorised horses according to their most used (≥ 50% scans) posture. Here, we focused particularly on backward ears, as this position has been previously shown to reflect chronically impaired welfare in horses [51].

#### Vertebral disorders

Evaluation of horses’ spines was performed by a licensed chiropractor with 20 years experience (H. Menguy), who was blind to the results of the observations performed during the behavioral tests, and did not know the horses beforehand. Manual palpation was performed from head to tail. Chiropractic approaches clearly address subclinical conditions (of special interest here) and licensed professionals have an expertise in the evaluation of joints and spinal related disorders [72, 73] which are efficient to detect back pain [74, 75]. This technique was chosen as radiographic imaging is limited by the thickness of the surrounding soft tissues [76]; ultrasonic and scintigraphic approaches remain difficult to apply in the field and on large samples of horses [76, 77].

Examinations were based on bony and soft tissue manual palpation to localise regions of vertebral stiffness based on spinal mobilisation and palpable areas of muscle hypertonicity [78, 79]. Comparisons of data from different practitioners have shown high agreement and therefore repeatability as well as correlations with electromyogram (EMG) data in other studies [37, 42, 44]. In the present study, a second examination by another practitioner (G. Bohn) gave 94% agreement. Examinations were performed outside the horses’ working hours, in their individual boxes. The horse was lightly restrained by one unknown (to the horses) experimenter (M. Hausberger) also blind to horse’s results in behavioral tests. Horses were classified by the practitioner as totally exempt, slightly affected (one slightly affected vertebral site) or severely affected (at least two severely affected vertebral sites). Data included also the number of affected vertebral sites.

#### Haematological data

At the same time as the morning cortisol sample was taken a blood sample was collected for each horse, between 08:00 and 09:00 and taken within the hour to a veterinary laboratory for analysis (Laboratoire Vétérinaire Départemental Ille et Vilaine, Rennes, France). Haematological data analysed were numbers of red blood cells (millions / mm^3^), white blood cells (million / mm^3^) and platelets (mille / mm^3^), percentages of neutrophils, eosinophils, basophils, lymphocytes, monocytes, hematocrit and haemoglobin (g/100ml), haemoglobin / hematocrit (g/100ml) and hematocrit / number of red blood cells per L (μm^3^). These analyses allowed us to identify horses with unusual levels of haematological parameters, such as anaemic animals. Norms provided by Laboratoire Vétérinaire Départemental Ille et Vilaine, Rennes, France and indicated in Table 1.

**Table 1:** Mean (±SEM) plasma cortisol (ng/ml) and fecal cortisol metabolite (FCM) (ng/g).

	Morning	Evening (day of work)	Evening (day of rest)
Plasma cortisol (ng/ml)	28.5 ± 1.4	15.2 ± 1.8	12.6 ± 1.1
FCM (ng/g)	-	5.0 ± 0.3	4.9 ± 0.4

#### Overall welfare summary

Based on our data, horses were further split into ‘compromised welfare’ and ‘normal welfare’ groups based on four measures; ear position, vertebrae disorders, neutrophil levels and anaemia. A ‘compromised welfare’ horse had at least 3 of the 4 measures associated with compromised welfare such as ears back greater than 50% of the time, severe vertebrae issues, neutrophil levels and/or anaemia outside the norms ‘Normal welfare’ horses had 0–1 of these compromised welfare measures. Previous work has shown that vertebrae disorders and haematological data showed no correlation while ears’ chronic backwards position seems to indicate an overall negative affect [42, 43, 67].

### Statistical analyses

Analyses were conducted using Statistica 13 (Dell Inc). T-tests were run to investigate circadian variations in cortisol levels. ANOVAs were used to calculate differences in fecal and plasma cortisol measures with day (rest vs work) when welfare indicators were divided into nominal categories (*e*.*g*. the horse spent more than 50% of its times with ears backward or not; the horse suffered from anaemia or not…). Pearson correlation tests estimated correlations between cortisol concentrations in plasma and fecal matter and occurrences or percentages of welfare indicators. p<0.05.

## Results

### Plasma cortisol and FCM concentrations

**Fig 1:**
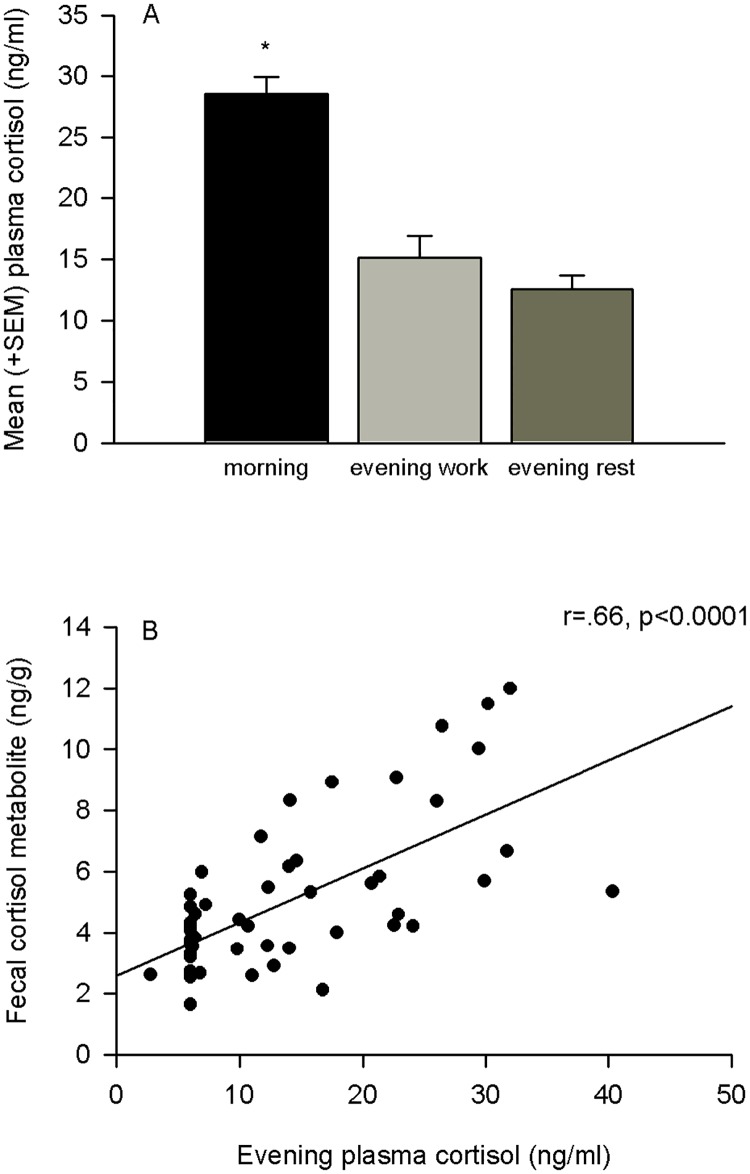
A) Mean (+SEM) plasma cortisol levels in horses. Plasma cortisol concentrations were significantly lower in the evening, both after a day’s work and after a day’s rest, than in the morning (p < 0.00001, N = 55–56). B) Average evening plasma cortisol levels and average levels of fecal cortisol metabolites were significantly correlated (r = 0.66, p<0.0001, N = 55). * denotes a significant difference.

In accordance to circadian variations previously reported in horses [61], plasma cortisol concentrations were significantly lower in the evening, both after a day’s work and after a day’s rest, than in the morning (N = 55–56, respectively t = 6.91, df = 55, and t = 8.7, df = 54, p < 0.00001 in both cases; Fig 1A). In addition, FCM concentrations did not significantly differ in relation to work and rest (N = 58, p > 0.3; Table 1). Average evening plasma cortisol levels (average of working and rest) and average FCM levels (average of working and rest) were significantly correlated (N = 55, r = 0.66, p<0.0001; Fig 1B) indicating that FCM measures may be used as an indicator of evening plasma cortisol levels. Neither age nor sex significantly influenced these effects (p>0.68).

### Cortisol concentrations in relation to welfare state indicators

#### Ear position

**Fig 2:**
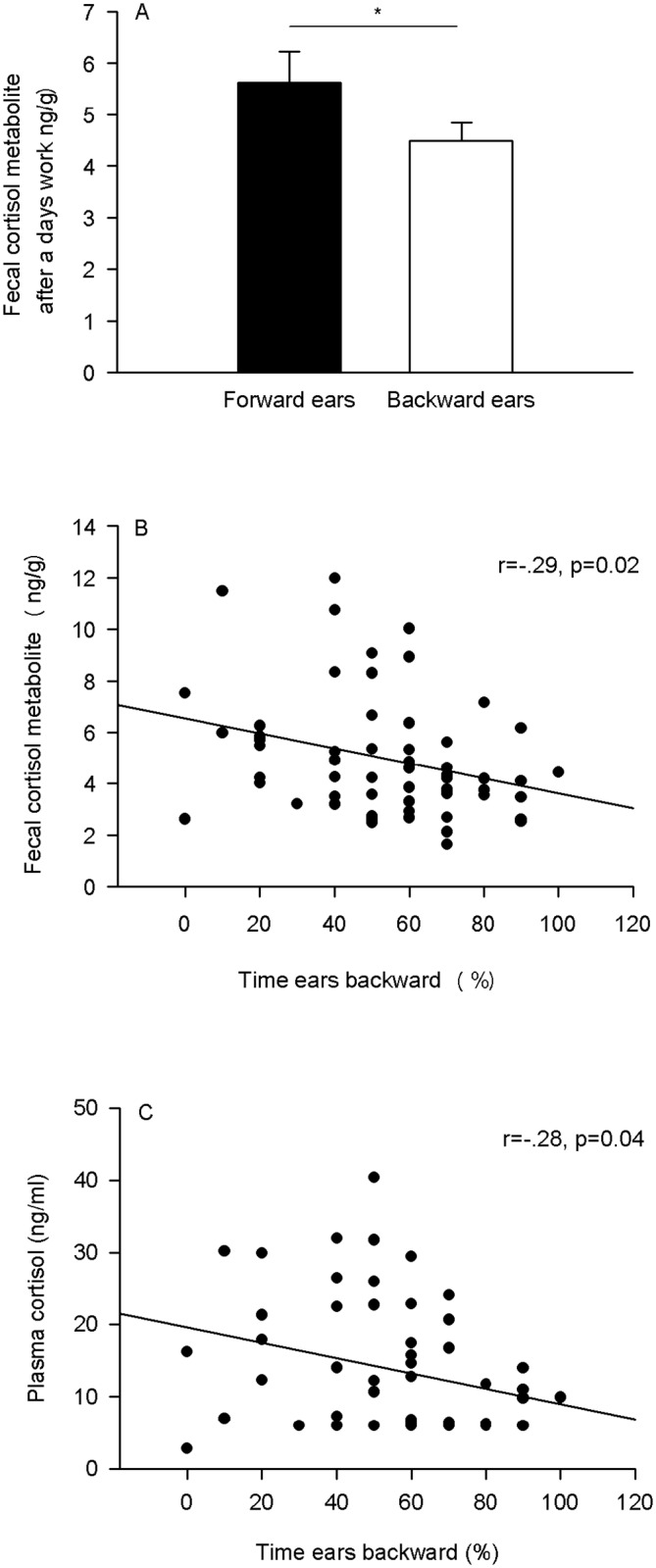
Mean (+SEM) average fecal cortisol metabolite levels after work as a function of ear position in horses. (A). Horses with mostly the backward ear position had significantly lower average fecal cortisol measures after work (p = 0.04). Time horses spent with ears in a backwards position was negatively correlated with average evening (B) FCM (p = 0.02) and (C) plasma cortisol measures (p = 0.04). (A) Forward ears N = 19, Backward ears, N = 31; (B) N = 59, (C) N = 55.

On average, the horses were observed with ears forward in 44.1% (± 21.8, 0–90) of scans, with their ears backward 53.4% (± 24.0, 0–100) of scans, and with ears in axial position 2.5% (± 5.4, 0–20) of the scans. 52% of horses showed ears backward in a majority of the scans (*i*.*e*. over 50% of the scans), and 32% showed ears forward in a majority of the scans. Horses with a backward ear position in a majority of the scans had significantly lower average fecal cortisol measures after a day’s work compared to horses with ears in the forward position (F(1, 48) = 4.48, p = .04, Forward ears N = 19, Backward ears, N = 31; Fig 2A). Moreover, percent of the scans horses spent with ears backwards was negatively correlated with average after work FCM and evening plasma cortisol measures (FCM N = 59, r = -0.29, p = 0.02; plasma cort, N = 55, r = -.28, p = 0.04; Fig 2B and 2C). There were no other significant differences between groups in ear position and FCM after a day’s rest or plasma cortisol levels on either the day of rest or work.

#### Vertebral disorders

Chiropractic examinations indicated, in accordance with the literature [45, 80–82], that most horses were severely affected by vertebral disorders (n = 40, 73%) (Chi-square test, N = 55, df = 2, χ^2^ = 38.44, p < 0.001). Only 27% of the horses were totally exempt or slightly affected by vertebrae problems (see also [39] for further details on vertebrae problems). Percentages of affected vertebrae per horse varied from 0 (totally exempt) to 88% of the spine ($\bar{X}\;=\;16.1\pm 19.1$).

**Fig 3:**
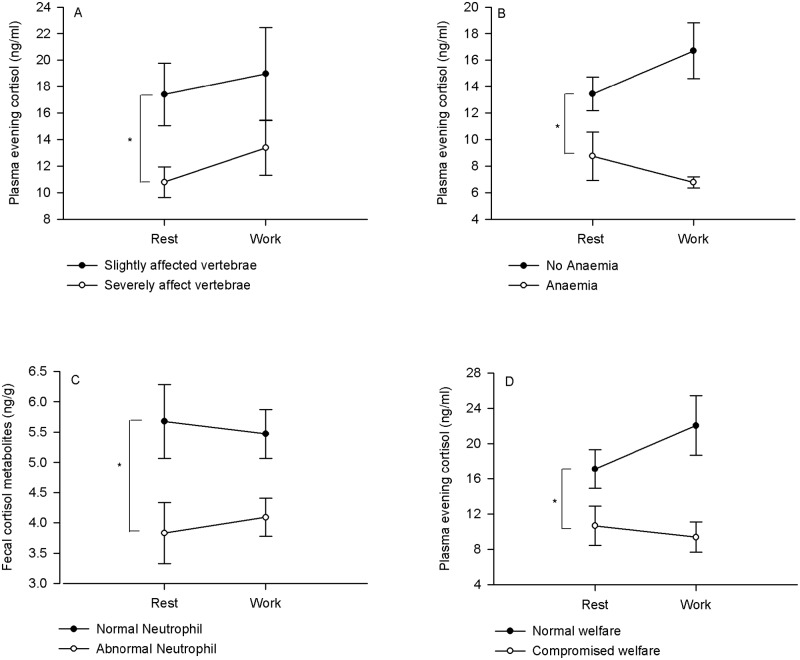
Mean (+SEM) cortisol measures related to measures of welfare in horses. A) Horses with severely affected vertebral problems had significantly lower evening plasma cortisol levels compared to totally exempt or slightly affected horses (p = 0.02; severely affected horses (n = 40) vs slightly affected horses (n = 15)). B) Anaemic horses had significantly lower evening plasma cortisol concentrations compared to non-anaemic horses (p = 0.02; anaemic horses = 10, non-anaemic horses = 45). C) Horses with levels of neutrophils outside the norm had significantly lower FCM levels compared to horses with normal neutrophil levels (p = 0.03; unusual neutrophils = 19, normal neutrophils = 36). D) Horses with ‘compromised’ global welfare had significantly lower evening plasma cortisol levels compared to ‘normal’ horses (p = 0.001; compromised = 13, normal = 17).). *denotes significant main effect, regardless of day of rest or work.

Severely affected horses (n = 40) had significantly lower evening plasma cortisol levels compared to totally exempt or slightly affected horses (n = 15), regardless of cortisol levels after a day’s work or rest (main effect of vertebral disorder severity F(1,53) = 5.34, p = 0.02; Fig 3A) but this same effect was not evident with measures of FCM (p = 0.3). There were no significant correlations between FCM or plasma cortisol measures and percent of vertebrae affected (p>.5).

#### Haematological anomalies

Our haematological data are summarised in Table 2. Of 55 horses that could be tested, ten horses (18%) suffered from anaemia (haemoglobin counts lower than norm) and 19 horses (35%) had neutrophils levels outside the norm.

**Table 2:** Haematological data of horses.

	Mean (±SD)	Observed range (*norms*)	% with unusual levels
Red blood cells (millions/mm^3^)	7.7 ± 0.9	6.3–10.6 (*6*.*5–12*.*5*)	0%
White blood cells (millions/mm^3^)	6.8 ± 1.7	3.1–11.7 (*5*.*5–12*.*5)*	0%
Neutrophils (%)	58.7 ± 11.8	26.0–81.1 (30–65)	42% (>)
Eosinophils (%)	2.0 ± 1.8	0.0–10.0 (0–5)	0%
Basophils (%)	0.2 ± 0.4	0.0–2.0 (0–3)	0%
Lymphocytes (%)	37.6 ± 11.4	18.0–70.0 (25–70)	11% (<)
Monocytes (%)	1.4 ± 0.8	1.0–5.0 (0.5–7)	0%
Hematocrit (%)	36.3 ± 4.5	34.0–43.0 (32–52)	0%
Haemoglobin (g/100ml)	12.4 ± 1.5	9.6–16.4 (11–19)	18% (<)
Haemoglobin/hematocrit (g/100ml)	34.7 ± 1.7	24.9–36.7 (34–39)	0%
Hematocrit/#red blood cells per L (um^3^)	46.7 ± 2.3	42.0–52.0 (34–58)	0%

Mean (±SD), range, and percent of horses with unusual levels: lower (<) and higher (>) than the norm. N = 55.

Anaemic horses had significantly lower evening plasma cortisol concentrations compared to non-anemic horses (main effect: F(1,53) = 5.8, p = 0.02; Fig 3B) and tended to have lower FCM levels compared to controls (p = 0.06). Horses with unusually high levels of neutrophils had significantly lower FCM levels compared to horses with normal neutrophil levels (main effect: F(1,52) = 5.1, p = 0.03; Fig 3C), regardless of whether FCM levels were after a day of work or rest. There were no other significant main or interaction effects between haematological anomalies and cortisol levels. Anaemia measures were significantly correlated with neutrophil levels (r = -.30, p = 0.027).

#### Global welfare

Further distribution of horses into ‘compromised’ welfare states (had 3 or 4 of the following: ears backwards in a majority of the observations (more than 50%), severely affected spine, neutrophils and/or anaemia above or below the normal range, and ‘normal’ welfare states (with 0–1 of the 4 measures) showed that horses with ‘compromised’ welfare had significantly lower average FCM and evening plasma cortisol levels compared to ‘normal’ horses (FCM—F(1,27) = 5.02, p = 0.03; plasma—F(1,28) = 13.1, p = 0.001; Fig 3D) and a day of work or a day of rest did not significantly affect this difference (ie. no significant interaction effect or main effect of day). Furthermore, there was no difference between ‘compromised welfare’ and ‘normal welfare’ horses in levels of plasma morning cortisol levels (p>0.5). (N = 13 compromised, N = 17 normal).

## Discussion

The findings of the present study, comparing horses’ baseline levels of cortisol and several welfare indicators, revealed that horses whose welfare was clearly compromised (as indicated by an unusual ears backward position, presence of vertebral problems or haematological anomalies, *e*.*g*. anaemia) also had lower levels of cortisol (and metabolites). This work extends our previous findings showing that withdrawn and immobile postures, indicators of depressive-like behavior in horses, are associated with lower plasma cortisol levels in horses [26]. The findings of the present study also show that there is a positive correlation between levels of plasma cortisol and fecal cortisol metabolites suggesting that FCM levels may be a comparable indicator of plasma cortisol levels in horses. Furthermore we showed that morning plasma cortisol levels are higher in horses, as expected [83].

### Low cortisol levels as an indicator of compromised welfare in horses

Our results expand earlier findings evidencing low or no change in cortisol levels after chronic training and racing of thoroughbred fillies [84] or chronic sub-optimal living conditions of horses [23, 24]. Our findings go further and show that cortisol could be lowered in cases of chronic compromised welfare as evidenced by behavior, anatomy, and haematology (present study). Low levels of glucocorticoids and/or decreased HPA reactivity after different types of chronic stressors have been reported in a number of species, including farm animals and humans [7, 8, 18–20, 85]. For example, chronic social isolation of calves results in lower baseline cortisol levels compared to group-housed controls [18]. Severely lame sheep experiencing chronic pain have lower plasma cortisol concentrations than did slightly lame and control sheep [86]. In humans, lower cortisol levels are associated with chronic stressors such as ‘burnout’ in teachers [85, 87] and chronic back pain [88]. In female European starlings, chronic stress not only induced a lower corticosterone level but also a lower reproductive success [21]. Altogether, these results support our findings in horses, where the lowest cortisol levels are linked with health-related indicators (vertebral problems and haematological anomalies) and postural indicators (previously shown to reflect health-related alterations and the presence of stereotypies [25],—indicators of compromised welfare.

Research to date in horses has shown that compromised welfare is associated with decreased cortisol levels, and/or blunted HPA axis activity [23, 24, 84]. Indeed the third phase of the “general adaptation syndrome” described by [89] is exhaustion. McEwen argued in favour of an “allostatic overload” mechanism in the case of chronic stress and development of pathologies in humans [90, 91] and this may be extended to other species. According to these authors, stress hormones play an important role in allostasis (maintaining stability, or homeostasis, through change), that is, the process of adaptation to events in daily life. When allostasis mediators, such as cortisol, are released in response to stressors or lifestyle factors such as diet, sleep—wake cycles, and/or exercise, they facilitate adaptation and are generally beneficial. However, when the stressor is sufficiently intense or prolonged, then the allostatic load (*i*.*e*. cumulative result of altered and sustained activity levels of the primary mediators) can increase dramatically until it reaches “allostatic overload”. Allostatic overload can then lead to a wear and tear of the system on the body and brain such that it no longer responds appropriately to the change in allostasis. The application of allostatic mechanisms to the animal welfare concept has been proposed recently to modify previous ideas related to homeostatic mechanisms alone [92]. Thus, it is possible that chronic confrontations with repeated welfare-related stressors in horses involve such an allostatic overload that there is a change in the physiological systems that normally respond to stressors, which may be reflected by low cortisol levels, haematologic anomalies, chronic pain and unresponsiveness (*i*.*e*. apathetic attitude). The mechanisms involved in this relationship between cortisol levels and welfare in horses remain to be investigated.

Although we have shown that low cortisol levels are associated with compromised welfare in horses, it has often been reported, in other species, that different types of chronic stressors, associated with general wellbeing, can also elevate glucocorticoid levels. These differences in effects may be due to the species studied, the type of stressor or welfare measure, duration of the stress, and techniques used to analyse glucocorticoid levels [4, 5, 93]. Reports in farm animals, laboratory rodents, and humans show that much of the variability of glucocorticoids levels under chronic stress is attributable to stressor features (type, controllability…) and individual features (experiential factors such as previous stressor experience) [7, 8, 19]. The factors that can challenge horses and, more globally, animal welfare are numerous (social and / or spatial and / or food-related deprivations, interspecific social conflicts with humans, chronic painful experience…). The horses studied here were stabled with restricted access to social contact and roughage, and tensed riding techniques (tight reins, high hands or amplified aids, strong control attempts on horses’ movements), which can alter welfare and the animal’s perception of its environment [35, 42, 43, 67]. Our population (only three riding schools) did not allow us to compare clearly the impact of all aspects of welfare, particularly living conditions and time working, on cortisol levels. However, future work aims to determine how living conditions such as living in stable social groups at pasture with minimal human interventions versus living in high density populations with few foraging opportunities, known to negatively affect horses’ welfare [94, 95], can affect horses global measures of well-being and cortisol levels. It is well documented that glucocorticoids can have an inverted-U shape relationship with cognition, as very low or high levels impair, whereas moderate elevations facilitate acquisition and retention of memory [96] and a similar inverted U-shape relationship may exist between between cortisol levels and global measures of welfare in horses. This could explain the lower learning abilities observed in stereotypic horses [33, 97] or even the altered attentional responses observed in horses with welfare alterations [66, 98].

### Correlation between plasma cortisol and fecal cortisol metabolite measurements

We also report a positive correlation between evening plasma cortisol levels and fecal cortisol metabolite concentrations in horses, extending our previous work [25]. Non-invasive sampling, via fecal matter, offers advantages over traditional invasive methods [2, 57]; Blood collection, which involves handling of animals, could itself be stressful and could confound the results [4, 99]. Consequently, use of a less invasive sampling method is advised. Salivary cortisol levels have been used in horses, however, contradictory results have been reported when compared with blood sampling, with reports of both correlations and no correlations between salivary and plasma cortisol [100, 101]. This discrepancy could be due to the limited sensitivity and specificity of assays of saliva samples and the role that corticosteroid binding globulins have in plasma cortisol levels [102, 103]: Plasma cortisol measurements are measures of total bound and unbound cortisol levels whereas salivary cortisol measures are measures of free levels of cortisol [102, 103]. Our results did show a clear positive relationship between evening plasma cortisol levels and FCM levels, regardless of a day of work or rest. It should be noted that FCM levels reflect an average level of circulating glucocorticoids during a time window with a species specific delay time [2, 3, 104], and thus, plasma cortisol levels may be advantageous at certain situations as they provide more direct measures of total cortisol levels at a given time point.

## Conclusions

The present study shows that horses living in compromised welfare conditions have low evening plasma cortisol levels and low levels of fecal cortisol metabolites (FCM). These lower levels of cortisol are associated with behavioral measures, such as ear positions (present study) and withdrawn posture [26], indicators of chronic pain (vertebral disorders), and haematological measures such as anaemia. Furthermore we show that plasma cortisol and FCM measures are correlated, thus providing an additional technique to analyse cortisol levels in horse populations. Future research aims to determine the extent to which factors influencing welfare, such as hours of work, living conditions (e.g. paddock versus pasture), early life factors, and human interaction, act as mediators of cortisol levels in horses.
